# Beyond Standard Parameters: Precision Hemodynamic Monitoring in Patients on Veno-Arterial ECMO

**DOI:** 10.3390/jpm15110541

**Published:** 2025-11-07

**Authors:** Debora Emanuela Torre, Carmelo Pirri

**Affiliations:** 1 Department of Cardiac Anesthesia and Intensive Care Unit, Cardiac Surgery, Ospedale dell’Angelo, 30174 Venice Mestre, Italy; 2 Department of Neurosciences, Institute of Human Anatomy, University of Padova, 35121 Padova, Italy; carmelo.pirri@unipd.it

**Keywords:** veno-arterial ECMO, extracoroporeal membrane oxygenation, hemodynamic monitoring, microcirculation, cardiogenic shock, echocardiography, arterial waveform analysis, precision medicine

## Abstract

**Background**: Hemodynamic management in veno-arterial extracorporeal membrane oxygenation (V-A ECMO) is inherently complex, as extracorporeal circulation profoundly alters preload, afterload, ventriculo-arterial coupling and tissue perfusion. This review summarizes current and emerging monitoring strategies to guide initiation, maintenance and weaning. **Methods**: A structured literature search was performed in PubMed and Scopus (1990–2025), including clinical studies, consensus statement and expert reviews addressing hemodynamic monitoring in V-A ECMO. **Results**: A multiparametric framework is required. Echocardiography remains central for assessing biventricular performance, aortic valve dynamics and ventricular unloading. Pulmonary artery catheterization provides complementary data on filling pressures, cardiac output and global oxygen balance. Metabolic indices such as lactate clearance and veno-arterial CO_2_ gap, together with regional oximetry (NIRS), inform the adequacy of systemic and tissue perfusion. Microcirculatory monitoring, though technically demanding, has shown prognostic value, particularly during weaning. Additional adjuncts include arterial pulse pressure, end-tidal CO_2_ and waveform analysis. Phenotype oriented priorities, such as detection of differential hypoxemia, prevention of left ventricular distension or surveillance for limb ischemia, require tailored monitoring strategies. Artificial intelligence and machine learning represent future avenues for integrating multiparametric data into predictive models. **Conclusions**: No single modality can capture the hemodynamic complexity of V-A ECMO. Precision monitoring demands a dynamic, phenotype-specific and time-dependent approach that integrates systemic, cardiac, metabolic and microcirculatory variables. Such individualized strategies hold promise to optimize outcomes, reduce complications and align V-A ECMO management with the principles of precision medicine.

## 1. Introduction

Veno-arterial extracorporeal membrane oxygenation (V-A ECMO) has become an essential rescue therapy for patients with refractory cardiogenic shock and severe circulatory failure, providing temporary support to systemic perfusion and oxygen delivery [1]. While its ability to stabilize critically ill patients is well established, the hemodynamic complexity introduced by this modality remains a major challenge for clinicians. By directly bypassing the native cardiopulmonary system, V-A ECMO profoundly alters preload, afterload, ventricular unloading and coronary perfusion [2]. These changes are further influenced by cannulation strategies, native cardiac function and the dynamic interplay between mechanical support and vasoactive therapies. In this setting, hemodynamic monitoring is not merely an adjunct but a cornerstone of patient management. Accurate assessment of cardiac performance, vascular tone and end-organ perfusion is indispensable to guide therapeutic decisions, optimize ECMO flow, prevent complications and tailor weaning strategies [3]. However, no single monitoring modality is sufficient to capture the multifaceted physiology of V-A ECMO. A comprehensive approach, integrating invasive blood pressure monitoring, echocardiography, advanced hemodynamic indices and novel technologies, is therefore required to translate physiological insights into clinical benefit. This narrative review aims to provide a critical overview of hemodynamic monitoring in V-A ECMO, highlighting the principles, strengths and limitations of available tools and discussing their practical implications for management of this high-risk population.

## 2. Materials and Methods

A structured literature search was conducted to identify studies addressing hemodynamic monitoring in patients supported with veno-arterial ECMO. The search was performed in PubMed and Scopus, covering the period from January 1990 to September 2025, using various combination of the terms “veno-arterial ECMO”, “hemodynamic monitoring”, “echocardiography”, “pulmonary artery catheter”, “microcirculation”, “differential hypoxemia”, “ECMO weaning” and “ventricular unloading”. The search included original clinical investigations, consensus statements and expert or systematic reviews focusing on patients undergoing V-A ECMO supports. Studies were retained if they explored one or more hemodynamic monitoring modalities relevant to ECMO initiation, maintenance or weaning and reported either quantitative or qualitative clinical outcomes. Animal studies, purely technical or engineering reports lacking hemodynamic data and non-peer-reviewed materials such as conference abstract were excluded. Titles and abstracts were initially screened for relevance, followed by full-text assessment to ensure eligibility. The reference lists of selected papers and major reviews were also examined to capture additional pertinent sources. Each included work was classified according to its primary domain of monitoring, echocardiographic, invasive, metabolic, microcirculatory or waveform-based assessment, so as to integrate complementary perspectives on cardiovascular physiology during ECMO. Given the heterogeneity of available data and the predominance of observational designs, no quantitative synthesis was attempted. Instead, a narrative approach was adopted to provide a critical appraisal of current evidence and highlight its practical implications for clinical management.

## 3. Relevant Sections

### 3.1. Hemodynamic Principles in V-A ECMO

V-A ECMO provides circulatory and respiratory support by diverting blood from the right atrium through a centrifugal pump, delivering it across a membrane oxygenator for gas exchange and reinfusing the oxygenated blood into the arterial system [4]. While this restores systemic oxygen delivery and perfusion, it profoundly alters native cardiovascular physiology. During peripheral femoro-femoral (F-F) V-A ECMO, the retrograde continuous flow from the arterial cannula interacts with antegrade pulsatile output of the left ventricle, creating a mixing zone in the aorta [5]. The position of this watershed depends on the balance between native cardiac output and ECMO flow and determines the relative distribution of oxygenated and deoxygenated blood to the coronary and cerebral circulation [6]. This artificial circulation disrupts normal ventricular–arterial coupling, leading to complex and sometimes deleterious effects on cardiac function. On the right side, ECMO drainage reduces right ventricular end-diastolic volume (RVEDV). In the setting of relatively fixed right ventricular contractility and pulmonary vascular resistance, this results in decreased right ventricular (RV) stroke volume [7]. On the left side, retrograde arterial flow increases mean arterial pressure (MAP) and systemic afterload. As ECMO flow rises, systemic pressure increases while arterial pulse pressure narrows, reflecting progressive suppression of left ventricular stroke volume. Concomitantly, the duration of aortic valve opening shortens and, in some cases, the valve may remain persistently closed, predisposing it to left ventricular distension, increased wall stress, pulmonary congestion and thrombus formation within the left ventricle (LV) cavity [8,9]. By contrast, the femoro-axillary (F-Ax) configuration delivers oxygenated blood in an antegrade fashion via the axillary artery. This approach ensures preferential perfusion of the supra-aortic branches and coronary circulation with fully oxygenated blood. The antegrade flow pattern also promotes a greater physiological pressure wave contour and reduces, but does not eliminate, the rise in LV afterload associated with ECMO support. Consequently, the hemodynamic interaction with the native LV is often more favorable compared with the F-F approach, supporting cerebral and myocardial protection [10]. In the central configuration, arterial return is established directly into the ascending aorta, providing fully antegrade systemic perfusion without the development of a mixing zone. This arrangement offers the most natural coupling between ECMO outflow and LV ejection. Nevertheless, LV afterload remains elevated, albeit to a lesser extent than in peripheral femoral cannulation. Central cannulation is typically reserved for the post-cardiotomy setting or as a bridge in advanced surgical strategies, reflecting its invasive nature and heightened risk of bleeding or infection [11,12].

Thus, although V-A ECMO can be life-saving by maintaining systemic perfusion and gas exchange, its impact on cardiac loading conditions requires careful monitoring and management. Balancing ECMO flow, ventricular unloading and afterload modulation is essential to mitigate adverse sequelae and optimize myocardial recovery.

### 3.2. Hemodynamic Challenges in V-A ECMO: Differential Hypoxemia and Pulmonary Congestion

V-A ECMO profoundly alters cardiovascular physiology and may give rise to complications that mandate dedicated monitoring. In the F-F ECMO the retrograde extracorporeal flow opposes native left ventricular ejection, creating a mixing zone in the aorta. When residual cardiac output originates from severely impaired lungs, the upper body may be perfused with desaturated blood, leading to differential hypoxemia (Harlequin syndrome) despite adequate systemic perfusion. Monitoring of right radial oxygen saturation, cerebral oximetry and targeted arterial blood sampling is essential to detect this condition [13,14].

At the same time, retrograde flow elevates systemic afterload, reducing left ventricular stroke volume and limiting aortic valve opening. Persistent closure predisposes it to LV distension, pulmonary congestion and intracavitary thrombosis. Continuous assessment of arterial pulse pressure, echocardiographic indices of ventricular size and valve dynamics and surrogate filling pressures enables timely recognition and initiation of unloading strategies [15,16].

#### 3.2.1. Optimization of Aortic Valve Opening and Pulsatility and Left Ventricular Decompression

Continuous monitoring of the aortic valve (AV) opening and arterial pulsatility provides crucial information on left ventricular (LV) distension and native ejection during V-A ECMO. Absent or intermittent AV closure is associated with blood stasis, increased LV end-diastolic pressure, pulmonary congestion and higher risk of intracavitary thrombosis [17].

Conversely, maintaining a regular AV opening reflects adequate LV decompression and partial recovery of native contractility. Hemodynamic strategies to promote AV opening aim to reduce LV afterload and optimize preload and contractility. Practical measures include avoiding excessive ECMO flow rates that completely suppress native ejection; cautious use of vasodilators (e.g., nitroprusside and milrinone) to decrease systemic vascular resistance; gradual titration of inotropes (e.g., dobutamine and Levosimendan) to enhance LV contractility and fine adjustment of volume status and PEEP to maintain optimal LV filling [15,18]. Hemodynamic monitoring parameters such as pulse pressure (PP > 15 mmHg), LVOT VTI ≥ 10–12 cm and rising end-tidal CO_2_ values during flow-reduction trials serve as non-invasive indicators of restored AV opening and improve native cardiac output. These metrics, integrated with echocardiographic evidence of forward ejection, support both ongoing management and readiness-to-wean decisions [19,20].

When these hemodynamic goals cannot be achieved despite optimization, LV venting becomes essential to prevent progressive distension, pulmonary edema and thrombus formation [16]. The indications for LV venting include persistently closed AV or minimal opening despite adequate inotropic support; elevated pulmonary artery wedge pressure (PAWP > 18–20 mmHg) or rising pulmonary congestion on chest imaging; echocardiographic evidence of progressive LV dilatation or spontaneous echo contrast indicating stagnant flow and risk of thrombosis; low arterial pulsatility (PP < 10–15 mmHg) or decreasing end-tidal CO_2_ despite stable ECMO flow, reflecting absent native ejection; development of pulmonary edema or evidence of refractory hypoxemia secondary to LV distension and lack of improvement in LV unloading after optimization of ECMO flow, volume status and pharmacologic support.

Several venting techniques are available, each with specific hemodynamic effects and risk profiles [21,22,23].

• Intra-aortic balloon pump (IABP): Reduces afterload and promotes intermittent AV opening by enhancing diastolic runoff. It is generally preferred when mild to moderate LV loading is present. Advantages include ease of insertion; however, its decompressive effect may be insufficient in cases of severe LV distension [24].
• Microaxial pumps (e.g., Impella devices): Actively unload the LV by draining blood from the LV cavity into the ascending aorta, thereby decreasing end-diastolic volume and wall stress. This method offers powerful unloading and improved LV recovery but carries risks of hemolysis, device migration and vascular injury [25,26].
• Trans-septal or atrial septostomy venting: Allows left-to-right shunting to decompress the left atrium, effectively reducing pulmonary congestion. It is minimally invasive but can result in right-sided volume overload or residual shunt post-decannulation [27].
• Apical LV venting, typically performed via a left mini-thoracotomy, allows for direct insertion of the cannula into the LV apex, which can be connected to the ECMO venous return or to a separate centrifugal pump (the latter configuration may function as a temporary LVAD-like system). This technique provides excellent unloading, but carries bleeding and myocardial injury risks [16,28].
• Right pulmonary vein venting, most often through via median sternotomy, is preferred in patients already undergoing open cardiac surgery. It ensures efficient decompression under direct visualization, but it is inherently more invasive and associated with postoperative bleeding and infection risks [29].

The choice of unloading or venting strategy should be individualized, balancing the degree of LV overload, the anticipated duration of support and the patient’s surgical risk, while integrating echocardiographic and hemodynamic findings to guide timely interventions.

#### 3.2.2. Differential Hypoxemia (Harlequin Syndrome)

Harlequin syndrome, also known as differential hypoxemia, is a distinctive complication of peripheral femoro-femoral V-A ECMO typically occurring in patients with severe respiratory failure such as ARDS, pneumonia or cardiogenic pulmonary edema. It results from competitive flow between retrograde oxygenated blood delivered by the ECMO circuit and the antegrade, poorly oxygenated blood ejected by the recovering native left ventricle. This interaction generates a dynamic mixing zone within the aortic arch, separating two perfusion territories: the upper body, including coronary and cerebral circulation, receives desaturated blood, whereas the lower body is perfused by oxygenated ECMO flow. As pulmonary gas exchange remains severely impaired but partial LV contractile function recovers, deoxygenated blood from native output reaches the aortic root, leading to a “two circulations” pattern characterized by upper body hypoxemia and cyanosis [30].

The incidence and severity of this phenomenon depend largely on the arterial cannulation site and on the balance between ECMO flow and native cardia output. With femoral cannulation, retrograde ECMO flow competes with antegrade LV ejection, often creating a distal aortic mixing zone and exposing the coronary and cerebral territories to hypoxemia; with axillary cannulation, antegrade oxygenated flow reaches the aortic arch and its branches, substantially reducing cerebral and coronary desaturation [10].

Early recognition is essential. Continuous monitoring should include right and left or femoral arterial oximetry, cerebral NIRS and arterial blood gas sampling (from both locations). A sustained gradient > 20% in oxygen saturation or a cerebral NIRS decline <50% suggest evolving differential hypoxia [6].

Management requires a structured, physiology-driven approach [13]:

• Optimize pulmonary gas exchange: increase FiO_2_ and PEEP, treat pulmonary edema or atelectasis, perform recruitment maneuvers and adjust ventilator settings to improve oxygenation of native LV output [6].
• Adjust ECMO flow: temporarily increase flow to move the mixing zone proximally, while avoiding complete suppression of LV ejection that may worsen stasis and distension [14].
• Transition to hybrid configuration (veno-arterial-venous VAV ECMO) in refractory cases, adding a return cannula to the right atrium, which enhances oxygenation of pulmonary circulation and equalizes systemic oxygen delivery [31,32].
• Central aortic cannulation: in post-cardiotomy or persistent cases, this ensures uniform antegrade perfusion to both coronary and cerebral arteries, providing definitive correction [13].

### 3.3. Volume Management and Hemodynamic Monitoring in V-A ECMO

**Figure 1:**
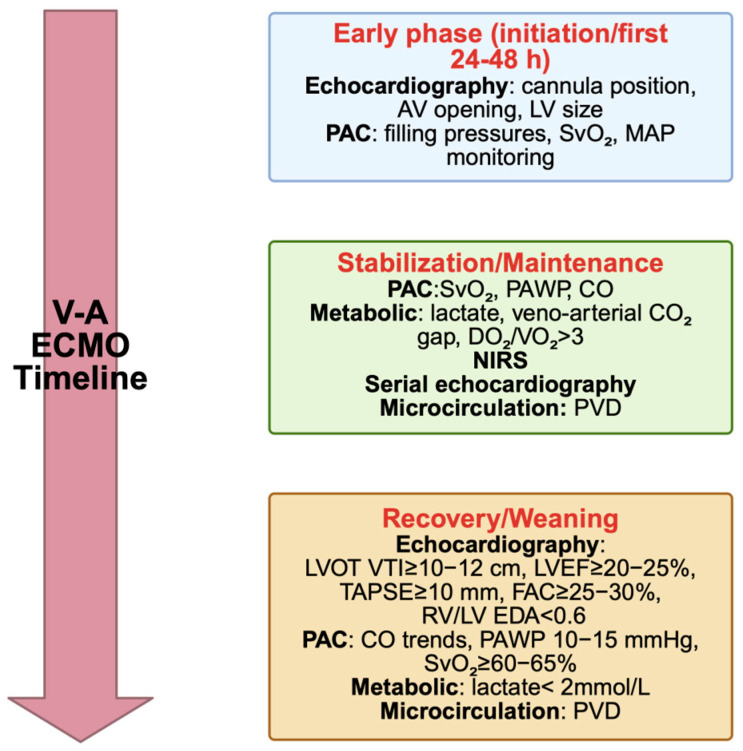
Time-dependent multiparametric hemodynamic monitoring in V-A ECMO. LVOT VTI: left ventricular outflow tract velocity time integral; LVEF: left ventricular ejection fraction; TAPSE: tricuspid annular plane systolic excursion; FAC: fractional area change; TDI S’: tissue Doppler imaging systolic velocity; RV/LV: right ventricle/left ventricle; PAC: pulmonary artery catheter; SvO_2_: mixed venous oxygen saturation; PAWP: pulmonary artery wedge pressure; CO: cardiac output; NIRS: near-infrared spectroscopy; PVD: perfused vessel density. Figure author generated using BioRender (49 Spadina Ave. Suite 200 Toronto ON M5V 2J1 Canada www.biorender.com).

Patients supported with V-A ECMO require meticulous volume management. Inadequate intravascular filing may cause suction events at the venous drainage cannula, impairing extracorporeal flow and compromising systemic support. Conversely, excessive positive fluid balance is consistently associated with poorer outcomes, underscoring the importance of early optimization of volume status [33,34]. Fluid management strategies should aim to facilitate left ventricular unloading and preserve end-organ perfusion and may include the use of renal replacement therapy when indicated [35]. Modern centrifugal pumps are inherently preload-dependent and afterload-sensitive, making serial assessment of filling conditions a clinical necessity. Hemodynamic monitoring should be multiparametric, integrating echocardiographic, invasive, metabolic and, when available, microcirculatory indices to provide comprehensive assessment. Such monitoring should be applied to all patients on V-A ECMO, yet it must also be personalized according to the underlying pathology and dynamically adapted to the clinical stage of support (initiation, maintenance or weaning). This individualized and time-dependent approach allows clinicians to ensure both circuit stability and effective systemic perfusion, while tailoring interventions to the patient’s evolving physiology (Figure 1).

### 3.4. Perfusion Monitoring in V-A ECMO: Clinical Parameters and Laboratory Indicators

Clinical examination remains a cornerstone in evaluating tissue perfusion in patients supported with V-A ECMO. Altered neurological status such as delirium or confusion (in non-sedated patients), cold and clammy extremities and oliguria often herald impaired systemic or regional perfusion. The skin, as an accessible vascular bed, provides valuable cues through indices including temperature gradients, mottling and capillary refill time (CRT). Both mottling and CRT have been validated as predictors of adverse outcomes in critically ill patients, though their applicability in ECMO patients remains unproven [36]. CRT and mottling scores may offer rapid, bedside insights into peripheral microcirculatory integrity, yet their sensitivity to central organ perfusion is limited and factors such as skin pigmentation can confound their interpretation [37]. Among laboratory parameters, serum lactate is widely recognized as a marker of tissue hypoxia and impaired oxygen delivery. Elevated levels during the early phase of V-A ECMO are strongly associated with increased mortality, while lactate clearance has emerged as a dynamic indicator of treatment response [38,39]. Nevertheless, hyperlactatemia is not specific to hypoperfusion: adrenergic stimulation, hepatic dysfunction and systemic stress may also contribute and impaired clearance frequently reflects microvascular dysfunction [40]. Mixed venous oxygen saturation (SvO_2_) and its surrogate, central venous oxygen saturation (ScvO_2_), provide further insight into the balance between oxygen delivery and consumption [41,42]. Although their role in shock resuscitation remains debated, low values consistently signal inadequate systemic oxygenation and have been correlated with mortality in V-A ECMO patients [43,44]. Continuous monitoring of pre-membrane venous saturation in the ECMO circuit offers a practical real-time surrogate for ScvO_2_, enabling early recognition of deteriorating perfusion [45]. Additional biochemical indicators may further refine the assessment of perfusion adequacy. Cardia biomarkers such as troponins and natriuretic peptides (BNP/NT-proBNP) provide indirect information on myocardial injury and ventricular overload, while renal markers, including urine output trends and emerging biomarkers such as NGAL, reflect the interplay between systemic perfusion and renal vulnerability [46,47,48]. Metabolic indices, particularly the venous-to-arterial carbon dioxide gap (Pv-aCO_2_), can serve as adjuncts to lactate in identifying impaired tissue perfusion and inadequate microcirculatory flow [49]. Finally, regional tissue oxygen saturation (rStO_2_), most commonly assessed by near-infrared spectroscopy (NIRS), offers a continuous, non-invasive window into cerebral and peripheral perfusion. Cerebral desaturation or interhemispheric differences have been associated with neurological complications, while asymmetries between the cannulated and non-cannulated limbs can indicate evolving limb ischemia. Though influenced by multiple systemic and technical variables, NIRS provides a useful adjunct for early detection of critical hypoperfusion and may guide timely interventions [50,51,52].

#### Monitoring and Prevention of Distal Limb Ischemia

Distal limb ischemia represents one of the most frequent and potentially preventable complications of peripheral V-A ECMO, occurring in up to 10–30% of patients undergoing femoral cannulation. The underlying mechanisms include obstruction of arterial inflow by retrograde femoral cannula, local vascular injury and inadequate collateral circulation. Hemodynamic and laboratory indicators of evolving ischemia include progressive reduction in limb temperature, pallor, mottling and increasing serum lactate or creatinine kinase levels. Bedside Doppler assessment of distal arterial flow and near-infrared spectroscopy (NIRS) monitoring (<40–50% or a >25% drop from baseline) are sensitive tools for early detection of regional hypoperfusion [53,54].

Preventive measures are centered on ensuring adequate distal limb perfusion at the time of ECMO implantation. The preferred approach is routine placement of a distal perfusion cannula (DPC) connected to the arterial limb of the ECMO circuit, typically inserted into the superficial femoral artery under ultrasound or fluoroscopic guidance. Continuous assessment of limb perfusion using NIRS, Doppler signals or serial clinical examination should be performed throughout support. In addition, direct monitoring of the DPC flow, either by inline flow sensor or pressure transducer, represents an effective preventive measure. A stable flow of ~100 mL/min indicates adequate distal reperfusion, whereas decreasing or absent flow should prompt immediate evaluation for kinking, thrombosis or malposition. If ischemic signs develop despite preventive measures, interventions include repositioning of downsizing of the arterial cannula, optimization of systemic perfusion pressure or revision of the distal perfusion line. In selected cases, conversion to axillary arterial cannulation can be considered to mitigate recurrent or severe lower limb ischemia. Early recognition and correction of distal ischemia are crucial to prevent irreversible tissue damage, compartment syndrome and subsequent limb loss [55,56].

### 3.5. Monitoring Mean Arterial Pressure in V-A ECMO

Mean arterial pressure (MAP) remains a fundamental parameter for ensuring adequate end-organ perfusion in patients supported with V-A ECMO. As the product of cardiac output (CO) and systemic vascular resistance (SVR), MAP can be modified by adjusting ECMO flow, optimizing native ventricular performance or administering vasoactive agents. Because total CO reflects both extracorporeal and native contributions, careful titration of these components is essential to maintain systemic stability. Although robust evidence defining optimal MAP targets in V-A ECMO is lacking, an initial goal of approximately 60 mmHg is widely accepted as a reasonable starting point [57]. Importantly, excessively high MAP values should be avoided, as they substantially increase left ventricular afterload, impair aortic valve opening and predispose individuals to pulmonary congestion and ventricular distension. Thus, management requires a delicate balance between ensuring adequate systemic perfusion and minimizing deleterious loading conditions on the failing heart [34]. Moreover, MAP alone cannot reliably reflect tissue-level oxygenation, as normalization of macrocirculatory parameters may coexist with persistent microcirculatory dysfunction [58]. For this reason, MAP monitoring should be integrated with complementary indices such as lactate dynamics, venous oxygen saturation, capillary refill, regional oximetry and Pv-aCO_2_.

#### 3.5.1. Arterial Pulse Pressure

The degree of pulsatility represent a valuable, real-time indicator of the balance between extracorporeal support and native cardiac ejection. Progressive narrowing of pulse pressure reflects diminished left ventricular stroke volume and reduced aortic valve opening, often signaling impaired ventricular unloading and the risk of pulmonary congestion or intracavitary thrombosis. Conversely, the presence of preserved pulsatility suggests residual contractile function and intermittent valve opening, which are favorable prognostic signs [59,60,61]. A pulse pressure <15 mmHg is indicative of cardiac native output < 1 L/min. Loss of pulsatility should therefore prompt further hemodynamic evaluation and may indicate the need for adjunctive unloading strategies.

#### 3.5.2. ETCO_2_

End-tidal carbon dioxide (ETCO_2_) reflects the interplay of pulmonary blood flow, ventilation and systemic metabolism. When these determinants are considered, ETCO_2_ can serve as a surrogate marker of native cardiac output during V-A ECMO [62]. Variations in ETCO_2_ in response to fluid resuscitation or vasoactive and inotropic therapy generally parallel directional changes in native cardiac output, although absolute correlation with cardiac output remains limited outside of very low values. An ETCO_2_ of below 14 mmHg has been shown to reliably indicate a cardiac output of less than 1 L/min in V-A ECMO patients [61]. Importantly, this threshold identifies a population at increased risk for LV overload and distension, warranting more comprehensive hemodynamic evaluation. Conversely, rising ETCO_2_ values, in conjunction with increasing arterial pulse pressure, when extracorporeal blood flow is held constant, provide readily accessible bedside indicators of myocardial recovery [63].

### 3.6. Role of the Pulmonary Catheter in V-A ECMO

The pulmonary artery catheter (PAC, Swan Ganz) provides comprehensive hemodynamic data, including measurements of cardiac output (CO) and right and left ventricular filling pressures: right atrial pressure and pulmonary artery wedge pressure (PAWP), pulmonary artery pressures and mixed venous oxygen saturation (SvO_2_) [64]. While the reliability of CO measurement obtained by thermodilution has traditionally been considered limited in V-A ECMO, since part of the native output is diverted into the extracorporeal circuit, the PAC nonetheless remains valuable for delivering critical information [65,66]. PAWP serves as surrogate for left atrial pressure and is a sensitive marker of left ventricular distension and pulmonary congestion. Central venous pressures (CVP), another parameter accessible via PAC, is often artificially reduced during V-A ECMO due to continuous venous drainage; conversely, persistently elevated CVP may indicate venous congestion, right ventricular dysfunction or impaired venous return but must always be interpreted within the context of ECMO flow dynamics, ventilatory settings, catheter positioning and vasoactive drug use [57,67]. Until recently, concerns about the inaccuracy of thermodilution-derived CO in extracorporeal settings were largely based on studies in V-V ECMO, which consistently showed significant overestimation due to indicator loss within the circuit [66,68,69]. This limitation was presumed to extend to V-A ECMO, where higher extracorporeal blood flow relative to venous return further compounds the error. However, a recent validation study by Levy et al. [70] suggested that continuous thermodilution with PAC may provide cardiac output measurements in V-A ECMO patients that are in good agreement with echocardiography-derived values, showing only a minimal positive bias and an acceptable percentage error. Notably, continuous thermodilution appeared to outperform bolus injection techniques by reducing recirculation artifacts and offering more reliable trend monitoring across different ECMO flow settings. Although these findings are encouraging and support the potential feasibility of PAC monitoring in this context, particularly when echocardiographic assessment is limited by suboptimal acoustic windows or arrhythmias, they should be interpreted with caution given the retrospective, single-center nature of the study. Consequently, while bolus thermodilution remains unreliable under full-flow V-A ECMO conditions, continuous thermodilution may represent a promising complementary tool to echocardiography and other monitoring modalities. Its greatest clinical utility is likely during phases of myocardial recovery and weaning, when accurate assessment of filling pressures and CO dynamics becomes most critical.

### 3.7. Echocardiographic Monitoring in V-A ECMO

Echocardiography remains the cornerstone of non-invasive hemodynamic monitoring in patients supported with V-A ECMO, allowing real-time assessment of cardiac loading conditions, ventricular function and the dynamic interaction between the heart and the extracorporeal circuit [71,72,73], (Table 1). It provides essential information from pre-implant evaluation to weaning, offering both diagnostic and prognostic insights.

Before ECMO initiation, echocardiography serves to quantify left and right ventricular function, identify contraindications such as severe aortic regurgitation and detect other structural pathologies including valvular disease, intracardiac thrombus or pulmonary embolism [74].

During cannulation, ultrasound ensures safe and accurate vascular access and correct placement of venous and arterial cannulae.

Post-cannulation imaging confirms appropriate tip positioning and identifies potential malposition, access insufficiency or pericardial effusion, all of which may critically influence circuit performance.

Throughout ECMO support, a structured and goal-oriented echocardiographic protocol should be applied. A baseline study at cannulation defines the initial hemodynamic state, followed by serial examinations, ideally daily or when major clinical changes occur, to reassess ventricular recovery, LV distension, valvular competence and pulmonary congestion. Key parameters include left ventricular size and ejection, aortic valve opening, LVOT VTI, degree of mitral regurgitation, right ventricular size and function (TAPSE, RV FAC) and pericardial effusion. These assessments are essential to detect complications such as LV overload or impaired ejection, which may require venting or flow adjustment [71,72,73].

Both trans-thoracic (TTE) and transesophageal (TEE) echocardiography are fundamental and complementary. TTE allows rapid bedside evaluation but is often limited by poor acoustic windows, particularly in ventilated or postoperative patients, due to mechanical ventilation, patient positioning or surgical dressings. TEE offers superior visualization of cardiac chambers, valves and cannula-related flow patterns, especially when TTE is suboptimal, though it requires expertise and carries a semi-invasive risk. Awareness of these technical constraints is crucial for reliable interpretation. In case of limited acoustic access, integration with invasive pressure monitoring, perfusion indices or pulmonary artery catheter data enhances diagnostic reliability [75,76].

### 3.8. Monitoring Microcirculation in V-A ECMO

V-A ECMO often restores systemic hemodynamic parameters such as blood pressure, cardiac output and SvO_2_, yet this does not necessarily translate into improved tissue perfusion [57,77]. The dissociation between macro- and microcirculation is a well-recognized feature of cardiogenic shock [78]. Hand-held video microscopy represents a promising bedside tool for direct assessment of microcirculation, though its use is limited to accessible regions such as the sublingual mucosa. Evidence indicates that sublingual microcirculatory alterations are prevalent in sepsis and cardiogenic shock and strongly correlate with mortality [79,80]. Early data in V-A ECMO patients suggest that impaired sublingual perfused vessel density (PVD) at cannulation predicts mortality, while failure to restore small vessel density or PVD within the first 24 h is also associated with adverse outcomes [79,80,81,82,83]. Furthermore, sustained values of total vessel density (TVD) and PVD during a 50% ECMO flow reduction were more sensitive and specific for predicting successful weaning than conventional echocardiographic indices [84]. Despite its potential, widespread adoption of sublingual microcirculatory monitoring remains hampered by the need for special equipment, operator expertise and time-intensive post-acquisition analysis. Further, multicenter studies are required to clarify its prognostic role and feasibility in routine V-A ECMO management.

### 3.9. Arterial Waveform Analysis

Arterial waverform analysis (APWA) systems, such as Vigileo, FloTrac (Edwards Lifesciences, Irvine, CA, USA), estimate cardiac output by deriving stroke volume from the arterial pressure curve and have been validated in perioperative settings despite operating without external calibration [85]. Their accuracy, however, markedly deteriorates in the presence of significant hemodynamic instability or vasomotor tone alterations. In V-A ECMO patients, profound fluctuations in vascular resistance and frequent loss of pulsatility due to extracorporeal flow further undermine the reliability of these devices [86,87]. Consequently, APWA is generally unsuitable during full-flow support or under high vasopressor requirements. Nonetheless, in carefully selected conditions such as during ECMO weaning, when extracorporeal flow is reduced, vasopressors doses are minimal and pulse pressure is relatively preserved, APWA may provide adjunctive information, though robust validation in this context remains warranted [57].

### 3.10. Monitoring of ECMO Blood Flow

The adequacy of V-A ECMO support is primarily determined by extracorporeal blood flow (ECBF), which must be tailored to the degree of residual native cardiac function. Patients with severely depressed myocardial function often require near-maximal ECMO support, whereas those with partial recovery can be managed with lower flow settings. Initial ECMO flow is generally set at 50–70 mL/kg/min, aiming to achieve a MAP > 60 mmHg and restore systemic perfusion [88]. Beyond systemic pressure, flow titration should be guided by markers of organ function, including hepatic, renal, pulmonary and neurological markers, underscoring the importance of interpretating ECBF values with clinical and laboratory indices of tissue perfusion. ECBF is influenced by both modifiable and static determinants. Modifiable factors include preload, afterload and pump revolutions per minute (RPM), while static factors are dictated by cannula diameter and length. In general, increasing RPM augments flow; however, when RPM is fixed, a decline in flow may reflect either reduced preload or elevated afterload. Preload limitation can result from bleeding, hypovolemia or impaired venous return, whereas afterload increase is typically due to high systemic vascular resistance, kinking of the cannula or oxygenator thrombosis. Careful bedside monitoring of these interactions is essential to avoid misinterpretation of flow changes [57]. From a physiological standpoint, the adequacy of ECBF must be interpreted within the balance of oxygen delivery (DO_2_) and oxygen consumption (VO_2_). DO_2_ is determined by the product of cardiac output (native + extracorporeal), hemoglobin concentration, arterial oxygen saturation and a constant factor. Adequate DO_2_ should generally exceed 330–350 mL/min/m^2^ in V-A ECMO patients, with a DO_2_/VO_2_ ratio > 3 regarded as a marker of sufficient systemic oxygen supply. When DO_2_ falls below this threshold, tissue hypoxia may occur even in the presence of seemingly adequate systemic hemodynamics [89]. VO_2_ can be estimated from mixed or central venous oxygen saturation (SvO_2_/ScvO_2_), veno-arterial CO_2_ gap and lactate clearance [90]. Persistent imbalance between DO_2_ and VO_2_, despite high ECMO flows, should prompt reassessment of hemoglobin concentration, oxygenator function and metabolic demands. From a practical standpoint, sudden reductions or oscillations in ECBF should prompt systematic evaluation of intravascular volume status, cannula position, circuit integrity and systemic vascular tone. Monitoring flow dynamics during ventilatory adjustment or vasoactive titration can also provide indirect information about ventricular loading conditions and the degree of interaction between native cardiac output and extracorporeal support. In peripheral ECMO, an additional clinical consideration is the risk of limb ischemia caused by femoral arterial cannulation. Placement of a distal perfusion cannula (DPC) is frequently recommended as a preventive measure. Flow through the DPC correlates positively with ECBF and may be assessed using an ultrasound flow meter. Although no universal cut-off exists, maintaining a DPC flow above 150 mL/min is commonly advocated for to reduce ischemic risk [91,92].

### 3.11. Rhythm Monitoring and Arrhythmia Management

Cardiac arrhythmias frequently occur during V-A ECMO and may precipitate hemodynamic instability, further impairing myocardial performance. They can result from myocardial ischemia, electrolyte imbalance, pharmacologic effects or occult bleeding. Continuous electrocardiographic monitoring is therefore mandatory to detect tachyarrhythmias, bradyarrhythmias or pacing failure. Ventricular fibrillation and sustained ventricular tachycardia are life-threatening and require immediate intervention with direct current cardioversion or antiarrhythmic therapy (typically amiodarone or lidocaine). Bradyarrhythmias or asystole, conversely, may necessitate temporary pacing, especially during weaning when native cardiac output contribution becomes critical. Supraventricular arrhythmias, such as atrial fibrillation with rapid ventricular response, should be managed with cautious rate control and correction of precipitating factors to avoid compromising ventricular filling and systemic flow. Early recognition and treatment of arrhythmias are integral to maintaining hemodynamic stability and optimizing myocardial recovery [57].

### 3.12. Integrative Hemodynamic and Echocardiographic Assessment for Weaning from V-A ECMO

Successful liberation from V-A ECMO requires a comprehensive, multiparametric evaluation that integrates systemic hemodynamics, ventricular function and tissue perfusion (Table 2). The “multiparametric evaluation” refers to the integrated interpretation of echocardiographic, invasive and metabolic indices. These parameters, such as LVOT VTI, LVEF, TAPSE, PAWP, MAP, SvO_2_ and lactate, provide quantitative thresholds which, when interpreted, guide flow adjustment, weaning readiness and follow-up decisions. Echocardiography remains the cornerstone, with a particular emphasis on LV recovery, aortic valve opening and LVOT VTI. Values of LVOT VTI ≥ 10–12 cm and left ventricular ejection fraction (LVEF) ≥ 20–25% are generally associated with higher likelihood of successful weaning [70]. Equally important is right ventricular (RV) assessment, as RV dysfunction frequently complicates cardiogenic shock and adversely affects outcomes. Favorable indices include a tricuspid annular plane systolic excursion (TAPSE) ≥ 10 mm, RVEF ≥ 25%, RV fractional area change (FAC) ≥ 25–30% and tissue Doppler S’ velocity > 6–10 cm/reflecting preserved RV systolic performance and balanced loading conditions [93,94,95]. Beyond isolated ventricular assessment, evaluation of biventricular interaction is essential, since the two chambers are mechanically and functionally coupled through the interventricular septum and the pericardial constraint. Excessive RV distension may shift the septum leftward, reducing LV preload and masking true LV recovery, while impaired LV ejection may secondarily increase pulmonary venous pressure and RV afterload. Hence, a stable septal position, coordinated recovery of both ventricles and preservation of RV-LV coupling represent key indicators of genuine myocardial recovery and readiness for weaning. Pulmonary artery catheter (PAC)-derived parameters provide complementary insights during flow reduction trials. Favorable trends include SvO_2_ ≥ 60–65%, PAWP around 10–15 mmHg without signs of congestion, stable or decreasing pulmonary artery pressures and rising native cardiac output when ECMO flow is reduced, preferably measured by continuous thermodilution [94,96]. Additional metabolic correlates of adequate perfusion are arterial lactate < 2 mmol/L with a declining trajectory, veno-arterial CO_2_ gap < 6 mmmHg and stable or improving NIRS values [97,98]. While they are still under investigation, indices such as the pulmonary artery pulsatility index (PAPi) have been suggested as potential predictors of RV recovery, though their applicability under V-A ECMO remains to be validated [99]. Beyond macro-hemodynamics, microcirculatory monitoring has emerged as a promising adjunct. Alterations in sublingual perfused vessel density (PVD) have been shown to correlate with weaning outcomes, often more accurately than conventional echocardiographic indices, underscoring the importance of integrating macro- and microcirculatory data [83,100]. Ultimately, no single parameter is sufficient; rather, weaning decisions should be based on a multiparametric framework combining echocardiographic indices of biventricular recovery, invasive hemodynamics, metabolic markers and microcirculatory monitoring to minimize the risk of premature decannulation or delayed support withdrawal.

This table summarizes proposed hemodynamic, echocardiographic and metabolic predictors of successful V-A ECMO weaning. Thresholds are based on observational data and expert consensus and should be interpreted within the clinical context using a multiparametric, patient-tailored approach. LVOT VTI: left ventricular outflow tract velocity time integral; LVEF: left ventricular ejection fraction; TAPSE: tricuspid annular plane systolic excursion; FAC: fractional area change; TDI S’: tissue Doppler imaging systolic velocity; RV/LV: right ventricle/left ventricle; PAC: pulmonary artery catheter; SvO_2_: mixed venous oxygen saturation; PAWP: pulmonary artery wedge pressure; CO: cardiac output; NIRS: near-infrared spectroscopy and PVD: perfused vessel density.

#### 3.12.1. Bridge Strategies and Hemodynamic Guidance After ECMO

The transition phase following V-A ECMO support represents a critical juncture in patient management, where decisions must be guided by both hemodynamic parameters and myocardial recovery trends. Depending on the degree and reversibility of cardiac dysfunction, patients may be candidates for one of several post-ECMO pathways: bridge to recovery, bridge to durable mechanical circulatory support (MCS), bridge to transplantation or bridge to decision.

• A bridge to recovery is feasible when sustained improvement in native cardiac output is demonstrated, typically reflected by stable systemic arterial pressure, MAP ≥ 65 mmHg without high inotropic support, LVOT VTI ≥ 10–12 cm, LVEF ≥ 20–25% and TAPSE ≥ 10 mm [93,94].
• Bridge to durable MCS or LVAD implantation should be considered in patients with insufficient myocardial recovery who remain dependent on ECMO flow to maintain perfusion or end-organ function. Key parameters include persistently low LVOT VTI (<10 cm), reduced pulsatility, elevated filling pressures (PAWP > 20 mmHg) or poor right ventricular reserve (FAC < 25%). Early transition to durable support is associated with improved outcomes compared with prolonged ECMO dependence [101,102,103].
• Bridge to transplantation represents an option for patients with irreversible myocardial failure but preserved end-organ function who are suitable candidates for heart transplantation. The decision to proceed toward transplant candidacy requires evidence of stable systemic perfusion, recovery of multi-organ function and the absence of fixed pulmonary hypertension which markedly increases the risk of early graft failure. Optimal pre-transplant hemodynamics are characterized by a mean pulmonary artery pressure (mPAP) < 25–30 mmHg, pulmonary vascular resistance (PVR) < 4 Wood units and a transpulmonary gradient (TPG) < 15 mmHg, ensuring that the donor’s right ventricle can adequately adapt to the recipient’s pulmonary circulation. Maintaining controlled afterload, adequate oxygenation and optimized ECMO flow during the bridging phase helps preserve a low pulmonary vasculature tone. In selected cases, transition to a temporary durable device (such as a paracorporeal LVAD or BiVAD) may be required to achieve longer-term stabilization and ensure transplant eligibility [104,105,106].
• For patients unsuitable for immediate recovery or transplantation, a bridge to decision approach allows ongoing stabilization while assessing neurological, renal and hepatic recovery to determine long-term candidacy for advanced therapies [107].

#### 3.12.2. Follow-Up and Long-Term Surveillance

Post-ECMO follow-up should be individualized according to the pattern of cardiac dysfunction, as post-decannulation trajectories differ substantially between left ventricular and biventricular failure. Patients with isolated LV failure often show gradual improvement of systolic function and may benefit from early echocardiographic reassessment within the first week after decannulation to confirm sustained recovery and exclude LV dilation or residual thrombus [74].

Conversely, patients with biventricular failure exhibit slower and less predictable recovery trajectories, requiring closer monitoring of RV size, function and pulmonary pressures, together with serial BNP or NT-proBNP levels [95].

In these cases, right heart catheterization or cardiac MRI may be indicated to evaluate persistent RV dysfunction or pulmonary hypertension. This phenotype-specific surveillance supports timely identification of incomplete myocardial recovery and guides transition to long-term mechanical support or transplantation when appropriate [108].

### 3.13. Artificial Intelligence and Machine Learning in Hemodynamic Monitoring

Artificial intelligence (AI) and machine learning (ML) are beginning to find applications in the management of patients supported with ECMO, although evidence remains limited and heterogeneous. Recent works have demonstrated the feasibility of using ML models to predict outcomes in V-A ECMO patients. For example, Wang et al. developed and externally validated a machine learning-based mortality prediction model, which outperformed conventional risk scores in identifying patients at high risk of early death following V-A ECMO support [109]. Similarly, ML approaches have been applied to pediatric ECMO cohorts to predict severe neurological injury, showing improved prognostic accuracy compared with traditional multivariable analysis [110]. In parallel, AI has been tested as a tool to enhance echocardiographic monitoring during ECMO. Chen et al. recently reported the use of AI-assisted algorithms to automate quantification of left ventricular function in pediatric ECMO patients, demonstrating performance comparable to expert cardiologists [111]. Beyond ECMO, broader advances in AI-driven echocardiography, including automated chamber segmentation, image quality assessment and functional quantification may hold particular promise for complex hemodynamic settings, where rapid, operator-independent evaluation is crucial [112]. Although these studies are preliminary, they suggest that AI could eventually support precision hemodynamic monitoring by integrating multiparametric data (e.g., echocardiography, PAC-derived pressures and perfusion indices) into predictive models. Large multicenter registries will be essential for training and validating such algorithms. At present, AI in ECMO should be regarded as an emerging adjunct, with its clinical role yet to be clearly defined.

## 4. Discussion

**Figure 2:**
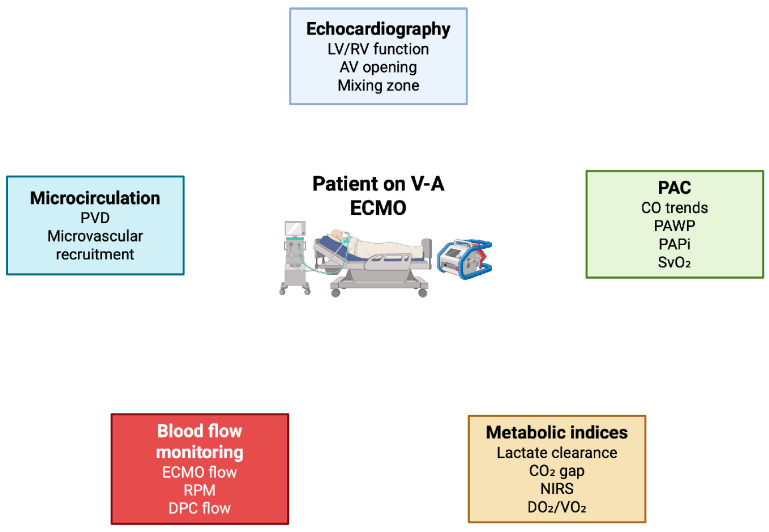
Patient-centered multiparametric monitoring in V-A ECMO. ECMO: extracorporeal membrane oxygenation; V-A ECMO: veno-arterial ECMO; PAC: pulmonary artery catether; CO: cardiac output; SvO_2_: mixed venous oxygen saturation; PAPi: pulmonary artery pulsatility index; LV: left ventricle; RV: right ventricle; LVOT VTI: left ventricular outflow tract velocity time integral; NIRS: near-infrared spectroscopy; DPC: distal perfusion cannula; RPM: revolutions per minute; PVD: perfused vessel density; DO_2_: oxygen delivery; VO_2_: oxygen consumption. Figure author generated using BioRender.

Hemodynamic monitoring in patients supported with V-A ECMO remains a major challenge, owing to the complex interplay between extracorporeal circulation, native cardiac output and tissue perfusion. The evidence reviewed in this study highlights that no single monitoring modality is sufficient to capture the full spectrum of hemodynamic alterations occurring during ECMO support. Rather, a multiparametric approach integrating echocardiography, pulmonary artery catheter-derived variables, metabolic indices and microcirculatory monitoring is required to guide clinical decision-making throughout the course of support, from initiation to weaning [113] (Figure 2).

In comparative terms, echocardiography remains the gold standard for evaluating ventricular loading and recovery, yet it is intermittent and operator-dependent [76].

Pulmonary artery catheterization, although invasive, allows for continuous assessment of filling pressures and mixed venous oxygen saturation, but cannot directly depict cardiac mechanisms [70]. Conversely, NIRS and lactate kinetics offer continuous and sensitive insights into tissue perfusion, though they lack specificity and may be influenced by extracardiac factors [39,49,57]. Combining these modalities therefore compensates for their individual weakness: echocardiography validates structural and functional change, invasive monitoring quantifies hemodynamic response and metabolic indices confirm adequacy of oxygen delivery at the microcirculatory level.

**Figure 3:**
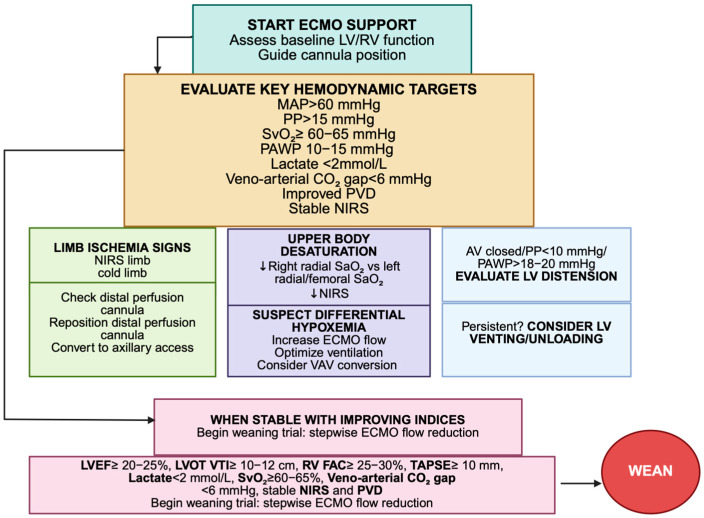
Practical algorithm for hemodynamic monitoring and management in V-A ECMO; V-A ECMO: veno-arterial ECMO; SvO_2_: mixed venous oxygen saturation; SaO_2_: arterial oxygen saturation; NIRS: near-infrared spectroscopy; PVD: perfused vessel density; MAP: mean arterial pressure; PAWP: pulmonary artery wedge pressure; LV: left ventricle; RV: right ventricle; PP: pulse pressure; VAV ECMO: veno-artero-venous ECMO; LVEF: left ventricular ejection fraction; RV FAC: right ventricular fractional area change; TAPSE: tricuspid annular plane systolic excursion; LVOT VTI: left ventricular outflow tract velocity time integral; upward arrow:increase; downward arrow: decrease. Figure author generated using BioRender.

A key concept emerging from this synthesis is the dissociation between macro- and micro-circulatory parameters [77]. Restoration of systemic blood pressure, cardiac output and SvO_2_ is rapidly achieved after ECMO initiation, yet tissue-level oxygen delivery and utilization may remain impaired, as shown by persistent lactate elevation, abnormal veno-arterial CO_2_ gradients or sublingual microcirculatory disturbances [82]. This discrepancy underscores the risk of relying on conventional systemic parameters alone and supports the incorporation of advanced bedside tools, such as hand-held video microscopy to provide real-time information on tissue oxygenation and microvascular recruitment. Another important consideration is that the relevance of monitoring modalities evolves over time. During the early phase, echocardiography and invasive monitoring are critical to confirm cannula positioning, exclude tamponade and assess ventricular distension. Later, as recovery ensues, serial echocardiographic indices of LVOT VTI, LVEF and RV performance, complemented by PAC-derived measures of SvO_2_, PAWP and CO trends, become central to assessing readiness for weaning [93,114]. Metabolic correlates such as lactate clearance, veno-arterial CO_2_ gap and NIRS add a further dimension by reflecting the adequacy of tissue oxygenation [39,49,98]. This time-dependent hierarchy illustrates why monitoring cannot be standardized but must be dynamically adapted (Figure 3).

From the perspective of personalized medicine, tailoring hemodynamic monitoring to the individual patient represents both the challenge and the opportunity of V-A ECMO. A patient with LV failure may benefit most from echocardiographic surveillance of LV size and function to guide venting and weaning strategies, while in predominant RV failure, indices such as TAPSE, FAC or PAPi are more informative for prognosis and therapeutic adjustment. In contrast, patients with biventricular failure require a truly integrated monitoring approach balancing right and left heart performance. In this setting, simultaneous evaluation of LVOT VTI, PAWP and left-sided congestion markers should be coupled with dynamic assessment of RV contractility and afterload. The interaction between both ventricles under ECMO flow may result in paradoxical responses, such as RV overload masking LV recovery, thus demanding close interpretation of coupled hemodynamic and echocardiographic data. Furthermore, in sepsis-related cardiogenic shock, systemic parameters may normalize despite impaired tissue oxygenation, making lactate clearance, DO_2_/VO_2_ balance and microcirculatory indices the most reliable markers of support adequacy. However, the widespread adoption of sublingual microcirculatory monitoring is currently limited by technical constraints, including the need for specialized equipment, trained operators and time-consuming offline analysis, restricting its availability to selected centers. Specific phenotype-oriented monitoring challenges further reinforce the need for tailoring strategies. Harlequin syndrome (differential hypoxemia), a complication of peripheral V-A ECMO, requires continuous integration of clinical, echocardiographic and oximetric monitoring. Invasive oximetry should include arterial blood sampling from the right radial artery, as it best reflects cerebral and coronary oxygenation while echocardiography allows for assessment of the mixing zone in the aorta. These tools are essential to promptly detect and correct maldistribution of oxygenated blood, guiding timely intervention such as ECMO flow redistribution, ventilatory adjustment or conversion to hybrid configuration (e.g., V-AV ECMO) [6]. Similarly, left ventricular distension represents a frequent and potentially deleterious complication, best recognized through echocardiographic evidence of absent AV opening, progressive LV dilation or worsening mitral regurgitation, often complemented by rising PAWP. Timely recognition through multimodal monitoring should trigger consideration of venting strategies, ranging from pharmacologic unloading to invasive approach such as septostomy, IABP or Impella support [84,115]. Finally, limb ischemia related to femoral arterial cannulation underscores the importance of integrated hemodynamic and vascular monitoring. DPC flow measurement, NIRS assessment of the limb and clinical examination must be systematically employed, since maintaining adequate peripheral perfusion is an essential component of the overall hemodynamic strategy in ECMO [50,116]. These examples demonstrate how clinicians must prioritize different parameters depending on the patient’s phenotype and evolving physiology. A critical comparison of current evidence also reveals the lack of consensus regarding thresholds or protocols for weaning. While parameters such as LVOT VTI ≥ 10 cm and LVEF ≥ 20–25% are widely cited, they perform variably across patient populations and are strongly affected by loading conditions [93,117].

Similarly, although continuous thermodilution has improved the accuracy of cardiac output estimation, its role under high-flow ECMO remains debated [70]. Emerging data on microcirculatory indices such as sublingual perfused vessel density (PVD) suggest superior predictive value for successful weaning compared with conventional echocardiographic metrics, but standardization and availability remain major barriers [81,82,83]. Comparative integration of these modalities, including macro, micro and metabolic modalities, represents the true evolution toward precision hemodynamic monitoring. This narrative contributes to precision critical care by framing hemodynamic monitoring in V-A ECMO not as a uniform checklist, but as a dynamic, patient-tailored strategy. The practical implication is that the success of ECMO support and weaning should be not judged solely by restoring systemic hemodynamics, but by achieving individualized targets of myocardial recovery, ventricular interaction and tissue oxygenation. Looking ahead, advances in artificial intelligence and machine learning hold promise for synthesizing the large volume of multiparametric data generated during ECMO support. Predictive models trained on such datasets may help identify patient-specific thresholds or dynamic patterns associated with recovery, complications or readiness for weaning, thereby operationalizing personalized monitoring in real-time. In conclusion, the current evidence supports a paradigm shift from standardized to personalized hemodynamic monitoring in V-A ECMO, where systemic, cardiac, invasive and microcirculatory and complication-focused parameters are interpreted in the context of each patient’s physiology and clinical trajectory. This tailored approach not only minimizes the risks of premature decannulation or unnecessary prolongation of support but also embodies the principles of precision medicine in critical care (Table 3).

Beyond physiological considerations, the economic and resource implications of advanced monitoring strategies deserve attention. To date, there is a lack of formal cost-effectiveness analyses specifically addressing monitoring strategies in patients supported with V-A ECMO. Nevertheless, the introduction of multimodal monitoring techniques, such as echocardiography-guided protocols, pulmonary artery catheterization, NIRS, arterial waveform analysis, invasive pressure monitoring, metabolic indices and microcirculatory assessment, inevitably entails additional resource utilization in terms of technology, operator expertise and procedural time. Evidence from surgical and perioperative settings has demonstrated that goal-directed hemodynamic optimization can be cost-effective, as it reduces postoperative complications, length of stay and overall hospital expenditures [118].

By analogy, precision-oriented monitoring in V-A ECMO may similarly enhance cost-efficiency by preventing complications (e.g., differential hypoxemia, left ventricular distension and limb ischemia), reducing unnecessary interventions and facilitating earlier weaning and ICU discharge. These potential benefits, together with improved patient selection and more timely recognition of myocardial recovery, highlight the need for future studies explicitly evaluating both economic and clinical outcome, in line with the principles of value-based critical care [119].

## 5. Conclusions

Hemodynamic monitoring in V-A ECMO represents a paradigm of complexity, where extracorporeal circulation profoundly alters cardiovascular physiology and conventional indices may be misleading. This review highlights that no single parameter is sufficient; instead, a multiparametric framework, encompassing echocardiography, invasive hemodynamics, metabolic indices and microcirculatory assessment, is essential to guide safe initiation, tailored management and successful weaning. Importantly, monitoring strategies must be individualized according to the patient’s phenotype and clinical trajectory, shifting the focus from standardized thresholds to patient-specific targets of myocardial recovery, ventricular interaction, tissue oxygenation and complication prevention. By embracing a personalized, dynamic approach, clinicians can minimize the risks of premature decannulation or unnecessary prolongation of support, ultimately improving outcomes and aligning V-A ECMO management with the principles of precision medicine in critical care.

## 6. Future Directions

The next frontier of hemodynamic monitoring in V-A ECMO will likely be shaped by technological innovation and precision medicine. First, advances in point-of-care imaging and automated echocardiographic quantification may allow for more standardized and reproducible assessment of biventricular functions and loading conditions. Second, integration of emerging biomarkers, including high-sensitivity troponins, natriuretic peptides and early renal injury markers such as NGAL, could provide complementary information on organ-specific recovery and guide therapeutic adjustments. Third, microcirculatory monitoring tools need to evolve from experimental hand-held video microscopy to more practical, real-time bedside technologies, enabling the routine incorporation of tissue-level perfusion indices into clinical decision-making. Finally, artificial intelligence and machine learning hold promise for synthesizing the vast amount of multiparametric data generated during ECMO support. Predictive algorithms may help identify dynamic thresholds associated with recovery, complications or readiness for weaning, thereby operationalizing patient-specific monitoring in real-time. In parallel, collaborative registries and multicenter trials will be essential to validate monitoring strategies and define evidence-based, phenotype-oriented algorithms. Taken together, these developments may transform hemodynamic monitoring from a largely observational practice into a proactive, predictive and personalized tool for optimizing ECMO care.

## 7. Limitations of the Literature

Despite a growing body of evidence, the literature on hemodynamic monitoring during V-A ECMO remains largely based on observational studies and expert consensus rather than randomized controlled trials. This predominance reflects the intrinsic challenges and ethical constraints of conducting interventional research in critically ill populations. As a result, much of the current understanding is derived from single-center experiences and registry analyses, which, although informative, may limit generalizability. In recent years, however, several comprehensive meta-analyses and systematic reviews have refined this evidence base, particularly in areas such as ventricular unloading, weaning predictors and comparative support strategies (Lim et al. [15], Lüsebrink et al. [23], Gandhi et al. [25], Hsu et al. [20], Charbonneau et al. [93]). These works provide an increasingly solid framework for clinical decision-making, while also highlighting the urgent need for prospective multicenter studies to validate monitoring algorithms and outcome-oriented thresholds in V-A ECMO management.

## Figures and Tables

**Table 1:** Practical echocardiographic assessment during V-A ECMO. LV: left ventricle; RV: right ventricle, TTE: trans-thoracic echocardiography; TEE: transesophageal echocardiography; LVOT VTI: left ventricular outflow tract velocity time integral; TAPSE: tricuspid annular plane systolic excursion; FAC: fractional area change; MR: mitral regurgitation; AV: aortic valve; SVC: superior vena cava; RA: right atrium.

Phase	Purpose of Echocardiography	Preferred Modality	Main Parameters	Limitations/Notes
Pre-implantation	Assess LV/RV function, exclude contraindications	TTE or TEE	LV and RV size and function, valvular competence, presence of thrombus	Limited by acoustic window in ventilated patients; TEE preferred in postoperative cases
During cannulation	Guide vascular access and confirm cannula positioning	TEE (preferred), TTE	Visualize guidewire and cannular tips in SVC/RA/aorta	Real-time ultrasound guidance to avoid vessel injury
During support	Monitor ventricular loading, LV distension, valve function, pericardial effusion	TTE or TEE	LVOT VTI, AV opening, MR, TAPSE, FAC, pericardial effusion	TTE limited by ventilation, dressings; TEE semi-invasive
Weaning trial	Assess myocardial recovery and readiness for decannulation	TEE or TTE	LVEF *≥* 20–25% LVOT VTI *≥* 10 cm Mitral S’ *≥* 6 cm/s RV performance adequate	Perform under low ECMO flow (<1.5 L/min). Requires stable anticoagulation
Post-decannulation	Detect vascular complications, residual dysfunction	TTE	Thrombus	Recommended follow-up after cannula removal

**Table 2:** Key hemodynamic and echocardiographic parameters proposed for weaning from V-A ECMO. IV: interventricular; LVOT VTI: left ventricular outflow tract velocity time integral; TAPSE: tricuspid annular plane systolic excursion; FAC: fractional area change; TDI: tissue Doppler imaging; RV: right ventricle; LV: left ventricle; PAPi: pulmonary artery pulsatility index; PAWP: pulmonary artery wedge pressure; NIRS: near-infrared spectroscopy.

Domain	Parameter	Proposed Threshold Weaning	Notes
Left ventricular function	LVOT VTI	≥10–12 cm	Reflects forward stroke volume; trend more relevant than single measure
	LVEF	≥20–25%	Low sensitivity alone; best if combined with LVOT VTI
Right ventricular function	TAPSE	≥10 mm	Associated with preserved RV systolic performance
	FAC	≥25–30%	Complementary to TAPSE; assesses global RV systolic function
	TDI S’ velocity	>6–10 cm/s	Reflects longitudinal RV systolic function
	RV/LV end diastolic area ratio	<0.6	Marker of balanced ventricular loading
Biventricular interaction	IV septal position and motion	Midline or mildly rightward; absence of paradoxical septal shift	Reflects interdependence; leftward shift indicates RV overload masking LV recovery
	Coupled trends in LVOT VTI and PAPi	LVOT VTI ≥ 10 cm with PAPi > 1.5	Suggests synchronized RV-LV recovery
Invasive monitoring (PAC)	SvO_2_	≥60–65%	Reflects global oxygen balance; declining SvO_2_ suggests inadequate CO
	PAWP	10–15 mmHg	Avoid congestion while maintaining adequate preload
	Native CO (continuous thermodilution)	Rising trend during flow reduction	Suggest ventricular recovery and readiness for ECMO weaning
Metabolic indices	Arterial lactate	<2 mmol/L, declining trajectory	Indicates adequate systemic perfusion and tissue oxygenation
	Veno-arterial CO_2_ gap	<6 mmHg	Reflects adequate tissue perfusion and oxygen utilization
	Cerebral NIRS	Stable or improving values	Proxy of cerebral oxygenation; should be integrated with systemic data
Microcirculation	Perfused vessel density (PVD)—sublingual	Improving/sustained values	Emerging tool; promising for predicting weaning success

**Table 3:** Phenotype-oriented strategies for patient-tailored hemodynamic monitoring in V-A ECMO.

Clinical Phenotype	Monitoring Priorities	Key Parameter/Tools
Predominant LV failure	Prevention of LV distension and optimization of unloading and weaning	Echo (LVEF, LV dimensions, AV opening, MR, LVOT VTI); PAC (PAWP, CO); markers of congestion; DO_2_/VO_2_ balance
Predominant RV failure	Assessment of RV systolic performance and pulmonary afterload	Echo (TAPSE, FAC, TDI S’, RV/LV EDA ratio); PAC (PAP, SvO_2_, PAPi); metabolic indices; response to vasodilators
Biventricular failure	Assessment of LV and RV performance	LVOT VTI, AV opening, RV and LV size, TAPSE, FAC, septal shift, pulmonary pressures, pulmonary congestion
Sepsis-related cardiogenic shock	Ensuring tissue-level perfusion despite normalized systemic hemodynamics	Lactate clearance, veno-arterial CO_2_ gap, NIRS, sublingual PVD (if available); DO_2_/VO_2_ ratio
Post-cardiotomy ECMO	Early recognition of myocardial stunning vs. irreversible dysfunction; bleeding and tamponade surveillance	Echo (pericardial effusion, biventricular function); PAC (filling pressures, CO); coagulation and lactate trends
Peripheral cannulation	Prevention of limb ischemia and monitoring of distal perfusion	Clinical exam; NIRS limb monitoring; DPC flow measurement (ultrasound or flowmeter); Doppler ultrasound
Harlequin syndrome	Detection of differential hypoxemia and distribution of oxygenated blood	Right radial arterial oximetry; echo (TEE to locate mixing zone); comparison with femoral PaO_2_/SaO_2_

This table highlights phenotype-specific priorities in hemodynamic monitoring during V-A ECMO. Although a multiparametric approach is necessary for all patients, the emphasis on certain modalities should be tailored according to predominant pathophysiology and clinical trajectory. LV: left ventricle; RV: right ventricle; LVEF: left ventricular ejection fraction; LVOT VTI: left ventricular outflow tract velocity time integral; AV: aortic valve; MR: mitral regurgitation; PAC: pulmonary artery catheter; PAWP: pulmonary artery wedge pressure; CO: cardiac output; DO_2_/VO_2_: oxygen delivery/oxygen consumption; TAPSE: tricuspid annular plane systolic excursion; FAC: fractional area change; TDI S’: tissue Doppler imaging systolic velocity; RV/LV EDA ratio: right ventricle/left ventricle end-diastolic area ratio; PAP: pulmonary artery pressure; SvO_2_: mixed venous oxygen saturation; PAPi: pulmonary artery pulsatility index; NIRS: near-infrared spectroscopy; DPC: distal perfusion cannula; TEE: transesophageal echocardiography; PaO_2_: partial pressure of oxygen in arterial blood; SaO_2_: arterial oxygen saturation.

## Data Availability

Not applicable.

## Abbreviations

The following abbreviations are used in this manuscript:

ECMO	Extracorporeal membrane oxygenation
V-A ECMO	Veno-arterial ECMO
LV	Left ventricle
RV	Right ventricle
TAPSE	Tricuspid annular plane systolic excursion
FAC	Fractional area change
PVD	Perfused vessel density
PAPi	Pulmonary artery pulsatility index
DO_2_	Oxygen delivery
VO_2_	Oxygen consumption
